# Impact of Vehicle Steering Strategy on the Severity of Pedestrian Head Injury

**DOI:** 10.3390/biomimetics9100593

**Published:** 2024-09-30

**Authors:** Danqi Wang, Wengang Deng, Lintao Wu, Li Xin, Lizhe Xie, Honghao Zhang

**Affiliations:** 1 State Key Laboratory of Automotive Simulation and Control, Jilin University, Changchun 130012, China; danqi_wang@csust.edu.cn (D.W.); xielizhe@126.com (L.X.); 2 School of Automobile and Mechanical Engineering, Changsha University of Science and Technology, Changsha 410114, China; dwg2113050004@163.com (W.D.); wlt052410@163.com (L.W.); 3 Hunan Province Key Laboratory of Safety Design and Reliability Technology for Engineering Vehicle, Changsha University of Science and Technology, Changsha 410114, China; 4 School of Automotive and Traffic Engineering, Jiangsu University, Zhenjiang 212013, China; xinli1126@163.com; 5 Key Laboratory of High Efficiency and Clean Mechanical Manufacture, School of Mechanical Engineering, Shandong University, Jinan 250061, China

**Keywords:** car-pedestrian collision, vehicle steering, pedestrian head injury

## Abstract

In response to the sudden violation of pedestrians crossing the road, intelligent vehicles take into account factors such as the road conditions in the accident zone, traffic rules, and surrounding vehicles’ driving status to make emergency evasive decisions. Thus, the collision simulation models for pedestrians and three types of vehicles, i.e., sedans, Sport Utility Vehicles (SUVs), and Multi-Purpose Vehicle (MPVs), are built to investigate the impact of vehicle types, vehicle steering angles, collision speeds, collision positions, and pedestrian orientations on head injuries of pedestrians. The results indicate that the Head Injury Criterion (HIC) value of the head increases with the increase in collision speed. Regarding the steering angles, when a vehicle’s steering direction aligns with a pedestrian’s position, the pedestrian remains on top of the vehicle’s hood for a longer period and moves together with the vehicle after the collision. This effectively reduces head injuries to pedestrians. However, when the vehicle’s steering direction is opposite to the pedestrian’s position, the pedestrian directly collides with the ground, resulting in higher head injuries. Among them, MPVs cause the most severe injuries, followed by SUVs, and sedans have the least impact. Overall, intelligent vehicles have great potential to reduce head injuries of pedestrians in the event of sudden pedestrian-vehicle collisions by combining with Automatic Emergency Steering (AES) measures. In the future, efforts need to be made to establish an optimized steering strategy and optimize the handling of situations where steering is ineffective or even harmful.

## 1. Introduction

With the development of science and technology, the emergence of vehicles brings convenience to people’s lives but also many hazards. Pedestrians, as the most vulnerable road users, are deeply affected by traffic accidents. The World Health Organization estimates that road traffic accidents worldwide cause about 1.3 million deaths per year, with more than two deaths per minute and more than 90 percent of these deaths occurring in developing countries. Since the invention of cars, more than 50 million people in the world have died in road traffic accidents [1]. In China, according to the National Bureau of Statistics, there are more than 60,000 casualties each year, with the pedestrian death rate exceeding 26% [2]. With the continuous increase in the penetration rate of civil vehicles and the ratio of car ownership, the burden of urban road traffic is gradually increasing, and the phenomenon of mixed traffic of people and cars on the road is serious, which leads to the increased frequency of vehicle-pedestrian collisions caused by sudden pedestrian illegal interference. Adopting traditional collision protection measures to passively reduce collision damage cannot achieve an active response to the collision process [3,4]. Therefore, a large number of researchers have begun to focus on intelligent driving, hoping to use vehicle active control to reduce damage or avoid collision situations. With the emergence of a series of intelligent driving technologies such as automatic emergency braking (AEB), automatic emergency steering (AES), and vehicle road coordination, the probability of accidents has been greatly reduced and vehicle travel efficiency has been improved [4,5,6]. Based on this background, this study aims to explore the impact of vehicle active collision avoidance measures on the severity of pedestrian injuries.

To reduce the mortality of pedestrians in traffic accidents, the severity of pedestrian injuries has been studied for decades. Huang et al. [7] predicted brain injury and injury severity in real traffic accidents through the in-depth study of brain impact response, injury, and tolerance in brain injury. Gabler et al. [8] used four different vehicle crash conditions to evaluate 15 existing kinematics-based indicators and predict the ability of strain-based brain response. Li et al. [9] conducted a detailed analysis of pedestrian head injuries in collision accidents based on a German database. Fahlstedt et al. [10] compare and assess head kinematics differences between TNO and THUMS in pedestrian accidents. Cai et al. [11] developed a finite element model of the head to study the biomechanical response of the head to windshield collisions and evaluate head injuries. They predicted the biomechanical response of head-to-windshield collisions at different impact speeds and windshield inclinations. Tian et al. [12] combined accident reconstruction methods with finite element analysis methods to study the damage mechanism caused by head landing after a pedestrian-vehicle collision.

The damage caused by pedestrian collisions with vehicles is complex and is influenced by collision speed, the shape of the front end of the vehicle, pedestrian physical signs (height, weight, gender, age, etc.), movement status (gait, walking speed, collision avoidance reaction, etc.), and the instant of physical contact with the ground [13,14,15,16,17,18,19]. Therefore, it is difficult to accurately predict and analyze pedestrian collision injuries. To better understand the injury mechanism and collision mechanism of pedestrians in collision accidents, researchers propose to link collision speed, vehicle front-end structure, pedestrian physical signs, and motion characteristics with injuries [20,21,22,23]. With the development of intelligent driving technology, in the face of sudden and emergency collision accidents, vehicles have a certain active braking function, so that through active braking measures, the occurrence of collision accidents or casualties caused by accidents can be minimized as much as possible [24,25]. Zou et al. [24,26,27] proposed a method of controlling vehicle braking to achieve control of pedestrian landing time to reduce contact damage between pedestrians and the ground in collision accidents. At present, few studies focus on the impact of vehicle active steering on injuries in the process of pedestrian-vehicle collision [25].

In nature, many animals avoid imminent threats by quickly changing the direction of their movement. Examples include the sideways jump of antelopes or the sudden turn of birds. This behaviour can be regarded as an emergency avoidance strategy in the animal kingdom. The emergency steering manoeuvres employed by smart vehicles can be seen as a technological mapping of such biological behaviours, reducing the risk of head injuries to pedestrians by mimicking this rapid response. It was found that when a vehicle evades at a steering angle of approximately 0.28 rad, it is effective in reducing pedestrian head injuries. This is similar to the way some animals reduce the risk of injury by adjusting their body posture and direction of movement when evading an obstacle. This mapping mechanism highlights how organisms can reduce injuries through adaptive behaviour, and we can apply this behavioural trait to the design of intelligent vehicles.

In addition, animals’ locomotion and avoidance strategies are often based on biomechanical principles of their body structure and locomotor capabilities. Similarly, intelligent vehicles can utilise similar biomechanical principles when taking avoidance measures. For example, the front-end structure and steering system of the vehicle can be designed to mimic the bending and twisting of the head or body of certain animals to achieve optimal collision avoidance. It was mentioned in the study that different collision locations and vehicle types lead to different injury outcomes, with MPVs resulting in higher injury values due to the specificity of the front-end structure. This is similar to the dynamic adjustments made by living organisms in complex environments. For example, different animals have different protection strategies when encountering an impact due to their size and body structure. They respond to the impact force with different body parts to reduce the risk of injury. These strategies can shed light on the optimal design of front-end structures of automobiles.

Compared with other simulation software for collision analysis, PC-Crash is an accident reconstruction tool widely used in traffic accident analysis. It has fast modeling and solving capabilities, but its accuracy in biomechanical simulation may not be as good as that of specialized biomechanical simulation software. LS-DYNA is a powerful finite element analysis tool capable of simulating complex nonlinear dynamic problems, which is suitable for high-precision biomechanical simulation. However, it may not always be the optimal choice for some application scenarios due to its complexity and high computational resource requirements. Similar to LS-DYNA, high-precision simulations in HyperWorks are often accompanied by high computational costs, requiring large computational resources and time. This may become a challenge when dealing with large-scale simulations. Therefore, MADYMO is finally chosen in this paper because it has excellent performance in biomechanical simulation, and it is especially suitable for analyzing pedestrian head injuries, and has a good balance of solution speed and accuracy, which can greatly improve computational efficiency. It is suitable for the complex vehicle-pedestrian collision scenario involved in this study. Based on this, this paper proposes to control vehicle steering to change the head fall position and pedestrian landing mode after the contact pedestrian-vehicle collision. Specifically, by adjusting the steering angle of the vehicle, it is possible to change the area where the pedestrian’s head comes into contact with the vehicle as well as the pedestrian’s position relative to the ground after a collision.

This control strategy aims to reduce head injuries to pedestrians, especially after an accident, by reducing the risk of direct head impact on the ground. This study provides a new optimization strategy for AES systems to reduce pedestrian head injuries by controlling the steering angle of the vehicle. This not only fills the gap in current research on automated driving strategies for pedestrian protection but also provides practical guidance for vehicle manufacturers and developers of intelligent driving technologies. The results of the study can be used to develop stricter road safety standards, especially for emergency response mechanisms for self-driving vehicles, to ensure the efficiency and reliability of intelligent vehicles for pedestrian protection. In addition, the results of this study are important for reducing pedestrian head injuries in traffic accidents, which in turn can help to improve the overall safety of road traffic.

Firstly, based on real accident cases, this article utilizes PC-Crash to reproduce accidents and extract collision parameters [21,28]. Secondly, to ensure the correctness of the model, multiple simulation experiments were conducted using MADYMO to establish appropriate numerical correlations between collision parameters and passenger injury standards. Further research was conducted on the impact of vehicle steering angle on pedestrian head injury [29,30]. Finally, based on the influence of steering angle, the position and state of human-vehicle contact are changed by actively controlling the vehicle’s steering angle, to reduce pedestrian head injury in frontal collision accidents.

Section 2 of this article effectively analyzes the collision model, collision parameters, head injury criteria, and collision model reconstruction validation, providing a parameter basis for later experiments. Section 3 presents the results of the impact of various collision parameters on head injury. Through a series of simulation studies, the relationship between the impact parameters and head injury is obtained. Section 4 is a discussion and analysis of the correlation between vehicle steering angle and head injury, to obtain the relatively optimal steering angle with minimal pedestrian head injury. The last section summarizes the experimental results and the shortcomings of this article.

## 2. Methods

**Figure 1:**
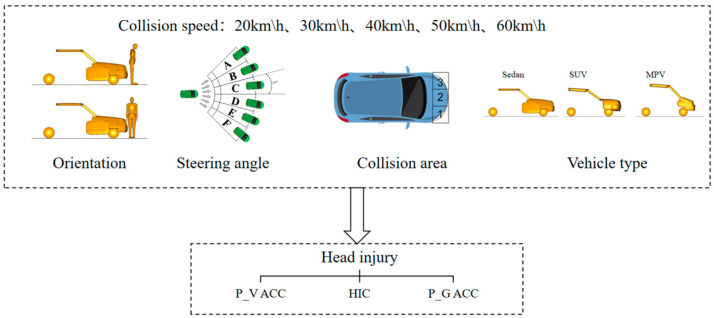
Collision parameters.

This study is based on real accident cases, using PC-Crash to reconstruct accidents, and using MADYMO to simulate collisions. By comparing with PC-Crash to restore accidents, the effectiveness of the MADYMO model is verified. Based on the effectively validated MADYMO model, orthogonal experiments were conducted to analyze the impact of collision parameters on head injury. In this calculation and analysis model, we use three vehicle models (sedan, SUV, MPV), and the collision speed varies from 20 km/h to 60 km/h an increment of 10 km/h [31,32]. This study used a 50th-percentile adult male for pedestrian models and defined two types of pedestrian-vehicle relative orientations and three collision areas at the front part of the vehicle to study the severity of pedestrian injury in collision accidents. To effectively verify the impact of vehicle steering angle on pedestrian injury severity, we simulated six collision scenarios under different steering angles (the 6 groups of steering angles are defined as angles A-F, where each group of steering angles defines A steering range of 15°, enabling the vehicle to steer from a range of −45° to 45° based on the horizontal line), compared and analyzed the pedestrian collision kinematics and injury prediction obtained, as shown in Figure 1.

### 2.1. Collision Model

**Figure 2:**
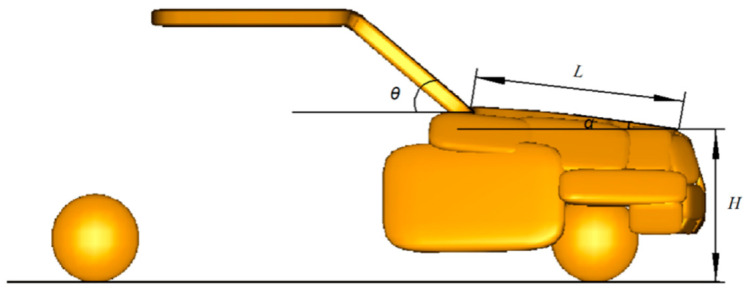
Front model (H: Hood front edge height; L: Hood length; *θ*: Windshield Angle; *α*: Hood Angle).

The simulations in this study were performed on a desktop computer equipped with an Intel^®^ Core™ i9-10900 CPU running at 2.80 GHz, 64 GB RAM, and an NVIDIA Quadro P2200 GPU. The entire simulation process, including the setup and running of 540 crash cases. Each simulation case took approximately 10 min to complete. Such computational resources and time requirements reflect the complexity of the model and the large number of simulations required to obtain reliable results. In each collision simulation, only a single collision parameter is changed to ensure that other collision parameters remain unchanged. The front of the vehicle is connected by multiple ellipsoidal combinations to simulate the shape of the front of three vehicle models (sedan, SUV, MPV), as shown in Figure 2. The front model consists of a bumper, hood, fenders, windshield, and four-wheel ellipsoids to approximate the exterior contour of the vehicle. The characteristics of these vehicles are that from sedans to MPVs, there are certain differences in the height, angle, and length of the hood leading edge [33]. Specifically, sedans are usually designed with greater consideration for pedestrian safety and have a relatively light body mass. Sedans usually have a lower body height and hood. When a pedestrian is struck, the head is more likely to strike the hood or front windshield. These areas usually have certain energy-absorbing designs, such as softer materials and shorter contact times that can mitigate head injuries. SUVs, on the other hand, are usually heavier than sedans and are characterized mostly by off-road capabilities and vehicle durability. SUVs are taller and have a higher hood position. Pedestrians, when struck, may directly impact the harder front of the vehicle or the hood, resulting in a greater impact to the head. Due to the higher hood of SUVs, pedestrians may be more likely to be lifted and roll to the ground; increasing the risk of a secondary collision. MPVs typically have a greater mass and tend to carry more passengers and cargo. In a pedestrian collision, the head is more likely to strike the vehicle’s A-pillar or harder front structure, resulting in more severe head injuries. The front-end parameters are shown in Table 1.

### 2.2. The Collision Parameters

This study simulates and analyzes the collision between pedestrians and vehicles with two different relative orientations. Simms et al. [34] conducted a study by defining five different relative orientations between pedestrians and vehicles to investigate the impact of the relative position and motion of pedestrians on collision kinematics and secondary injuries in pedestrian-vehicle collisions.

**Figure 3:**
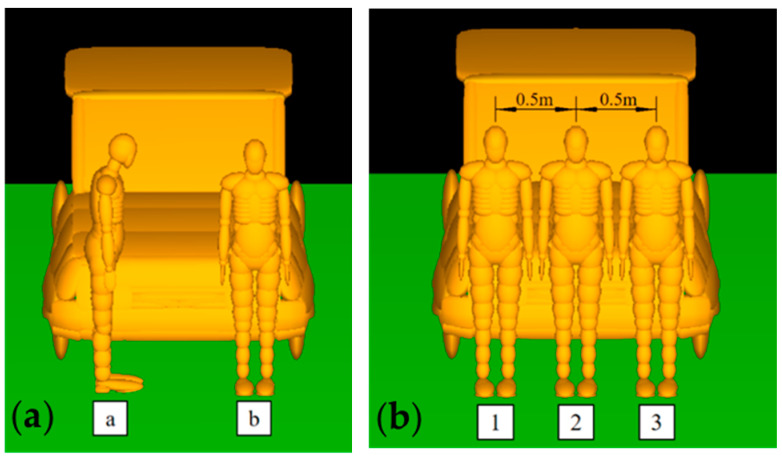
(**a**) Relative orientation of people and vehicles: a. Pedestrian side to vehicle; b. Back to the vehicle. (**b**) The relative position of people and vehicles.

Based on this research, this article divides the relative orientation of pedestrians and vehicles into two categories: pedestrian side to vehicle “a” and pedestrian back to vehicle “b”. As shown in Figure 3. To better define the contact location between pedestrians and vehicles, Wang et al. [35] divided the vehicle into 16 collision zones to better study the deformation characteristics of each part during vehicle collisions. In this study, the front part of the vehicle was analyzed for collisions. Therefore, the front part of the vehicle was divided into three regions: the left side, right side, and middle collision regions. By defining the relative orientation of the pedestrian and the vehicle, as well as dividing the front collision area of the vehicle into different regions, it is possible to define the relative motion state between the pedestrian and the vehicle before the collision occurs. As shown in Figure 3. To simulate a wide range of collision conditions, five different collision speeds were considered for each pedestrian position and vehicle type. Collision simulations were conducted for these five vehicle speeds ranging from 20 km/h to 60 km/h to replicate the typical range of collision speeds in pedestrian accidents [36,37]. Due to the lower survival rates for pedestrians in high-speed collisions, this study did not discuss high-speed collisions.

### 2.3. Steering Angle

As the objective of this study is to determine whether vehicle active steering has a significant impact on pedestrian injuries in collision accidents, the steering angles of the vehicles were numerically defined in this study. By analyzing pedestrian-vehicle collision accidents, the steering angles of the vehicles were divided into six different angles in this study. In MADYMO collision simulation, vehicle steering control during pedestrian-vehicle collisions is achieved by changing the vehicle’s lateral velocity and the angular velocity of rotation around the hinge joint. For example, at a collision speed of 40 km/h, the vehicle undergoes a sequence of steering angles A to F. In MADYMO software, the vehicle’s longitudinal velocity Vx of the vehicle is set to 11.11 m/s, the lateral velocity Vy is set to −1.8 m/s and the angular velocity w for rotation around the hinge is set to −0.9 rad/s. To achieve the completion of vehicle steering angle A during the collision with a pedestrian, the lateral velocity and angular velocity for rotation around the hinge joint for steering angles B to F are shown in the Table 2. To ensure consistency in variables, the angle parameters for other collision speeds are also set according to this table.

### 2.4. Injury Criteria

When assessing the risk of pedestrian injuries, particular emphasis is placed on evaluating head injuries. We use the Head Injury Criterion (HIC) for damage analysis based on head kinematics. By simulating the collision process and combining it with HIC standards, the severity of pedestrian head injury can be analyzed [38,39,40]. Research shows that the maximum head rotation acceleration is closely related to brain injuries, and injury criteria based on head rotation kinematics have been proposed to assess the risk of head injuries. However, the application of these standards is still limited to the research field. In comparison, the maximum resultant linear acceleration of the head’s center of gravity (CG) and HIC based on linear acceleration (acceleration magnitude and duration) have been used for many years in vehicle safety standards [38]. HIC estimates the severity of head injury by correlating the effective deceleration of the head during the impact process. The mathematical definition of HIC is:

$$\mathrm{HIC}={\left\{\left(t_{2}-t_{1}\right){\left[\frac{1}{t_{2}-t_{1}}\int_{t_{1}}^{t_{2}}\left(\frac{a\left(t\right)}{g}\right)\mathrm{dt}\right]}^{2.5}\right\}}_{\max}$$

where, *g* represents 9.81 m/s^2^, *a*(*t*) represents head synthesis acceleration (m/s^2^), *t*_1_ and *t*_2_ represent moment (s) of maximum HIC.

### 2.5. Experimental Validation

**Figure 4:**
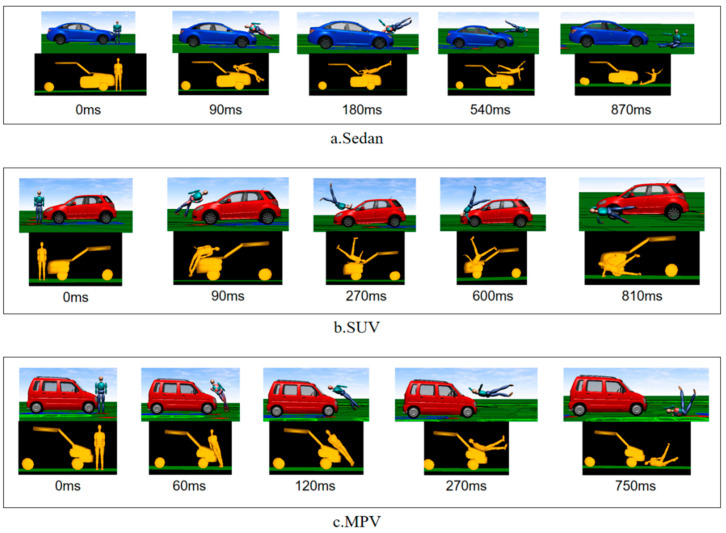
(**a**) Comparison between PC-Crash and MADYMO reconstruction for Sedan; (**b**) Comparison between PC-Crash and MADYMO reconstruction for SUV; (**c**) Comparison between PC-Crash and MADYMO reconstruction for MPV.

This study focuses on conducting collision injury analysis using simulation software. Firstly, based on the existing real-world collision cases involving turning vehicles and pedestrians in the laboratory, a selection is made of three vehicle types (sedan, SUV, and MPV) that exhibit turning characteristics when colliding with pedestrians. The selected accident cases are reconstructed using PC-Crash 12.0 to obtain the kinematic parameters of the vehicle’s steering and the pedestrian’s collision motion state during the collision process. Based on the parameters of the pedestrian-vehicle collision accidents (as shown in Table 3), a multi-body model of the pedestrian-vehicle collision is reconstructed using MADYMO simulation software. The MADYMO reconstructed model is then compared and verified against the PC-Crash reconstructed scenario, as shown in Figure 4.

## 3. Results

### 3.1. The Impact of Collision Velocity on Injury

**Figure 5:**
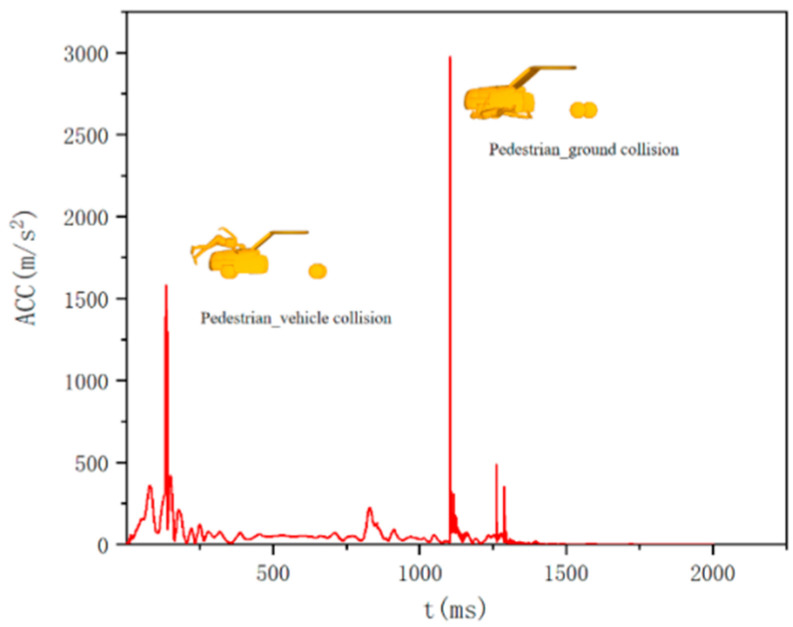
Head composite acceleration curve.

**Figure 6:**
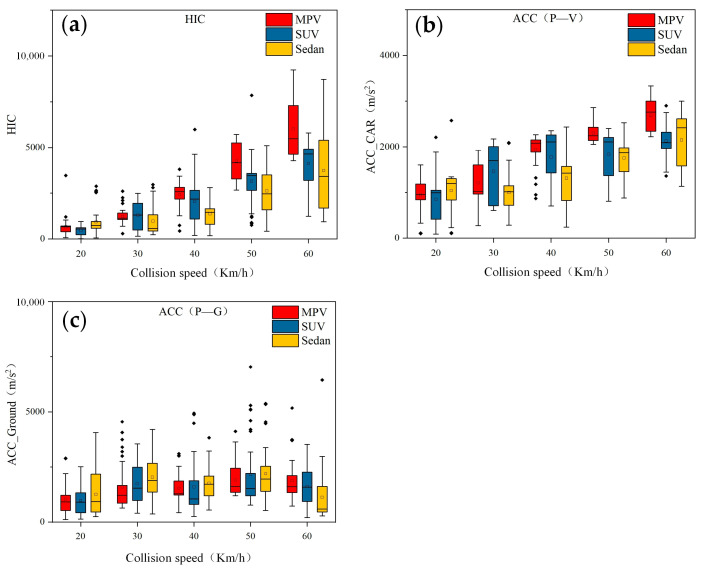
HIC and ACC box plots caused by three vehicle models at different speeds: (**a**) HIC; (**b**) peak human-vehicle acceleration; (**c**) peak human-ground acceleration.

In the face of a pedestrian-vehicle collision accident, pedestrians experience two major impacts, as shown in Figure 5. The first time is a pedestrian-vehicle collision, and the second time is a pedestrian landing, where landing will cause greater pedestrian damage. Figure 6 shows the Head Injury Criterion (HIC) values of the Hybrid III 50th percentile male dummy model used in this study, as well as the peak values of the pedestrian-vehicle acceleration and pedestrian-ground acceleration at different collision speeds. Where the two plots of peak acceleration have different scale ranges, with the peak values of the pedestrian-vehicle acceleration having a scale range of 0–4000 and the pedestrian-ground acceleration having a range scale of 0–10,000. Each boxplot contains 540 data points, which are the results of simulation and modeling using three different target vehicle models and varying collision speeds.

It can be observed that as the collision speed of the vehicle increases, the Head Injury Criterion (HIC) values for pedestrian head injuries also increase accordingly. Additionally, the differences in head HIC values obtained from simulation using three different vehicle models (Sedan, SUV, and MPV) become more pronounced at higher speeds (over 40 km/h). When the speed is 60 km/h, it can be observed that the MPV causes the highest level of head injury to pedestrians, while the difference between sedans and SUVs is relatively small.

For lower speeds (below 30 km/h), the difference between the three vehicle models is not significant. For collision acceleration, there is a large difference in the peak acceleration caused by the collision between people and vehicles. The peak acceleration also increases with the increase of collision speed. The peak value caused by MPV is higher than that of the other two models, which is similar to the effect of head injury HIC. Overall, in this study, the MPV has the greatest impact on pedestrian head injuries, among the three vehicle models, followed by SUVs, and sedans have the least impact. Furthermore, as the collision speed increases, the impact on the head also increases.

### 3.2. The Effect of Steering Angle on Injury

**Figure 7:**
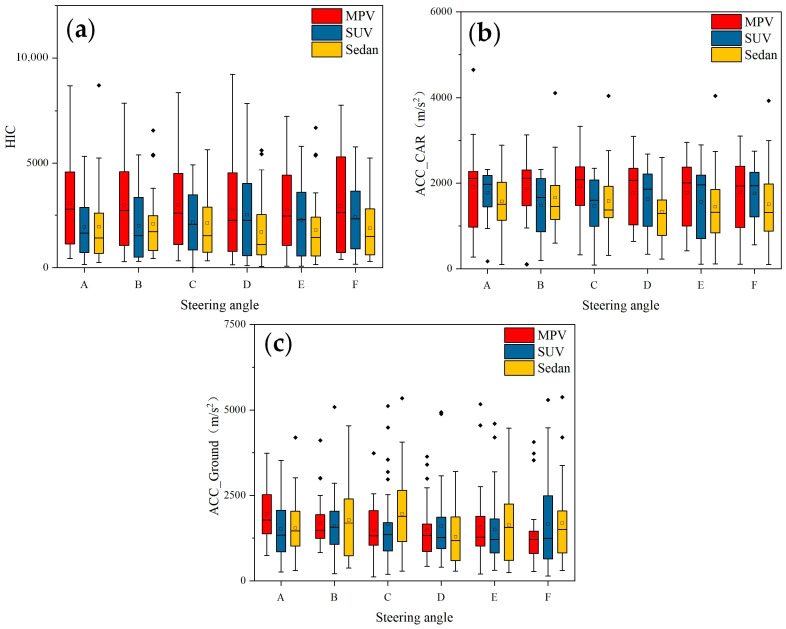
HIC and ACC box diagram caused by three vehicle models under different steering conditions: (**a**) HIC; (**b**) peak human-vehicle acceleration; (**c**) peak human-ground acceleration.

**Figure 8:**
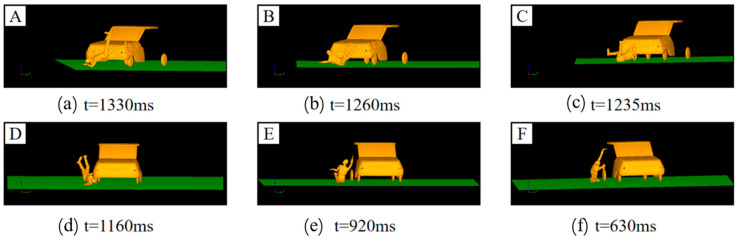
Passenger landing situation in a 40 km/h car collision with different steering angles: (**a**) Steering Angle-A landing time t = 1033 ms; (**b**) Steering Angle-B landing time t = 1260 ms; (**c**) Steering Angle-C landing time t = 1235 ms; (**d**) Steering Angle-D landing time t = 1160 ms; (**e**) Steering Angle-E landing time t = 1033 ms; (**f**) Steering Angle-F landing time t = 1033 ms.

Figure 7 presents the box plots of pedestrian-vehicle acceleration, pedestrian-ground acceleration, and head injury criterion (HIC) values for different vehicle turning angles in a car-pedestrian collision. During a vehicle-pedestrian collision, turning the vehicle will result in more lateral collision force and acceleration on the pedestrian compared to a non-turning collision. Simulation analysis shows that turning also has a significant impact on the pedestrian’s landing posture, as shown in Figure 8. When a car traveling at 40 km/h collides with a pedestrian on the right front of the vehicle, simulation results show that the pedestrian’s landing time changes with the vehicle’s turning angle. When the pedestrian-vehicle collision position is in the same direction as the vehicle’s turning direction, the pedestrian’s landing time increases with the increase of the vehicle’s turning angle (Figure 8a–c). When the collision position is opposite the vehicle’s turning direction, the landing time decreases with the increase of the turning angle (Figure 8d–f).

This phenomenon occurs because when the collision position is in the same direction as the turning direction, the pedestrian is impacted as the pedestrian comes into contact with the front part of the vehicle and moves together with the vehicle for a longer time. When the collision position is opposite to the vehicle’s turning direction, the pedestrian is located at the edge of the vehicle’s front. After being impacted, the pedestrian comes into less contact with the front part of the vehicle; resulting in the pedestrian landing directly.

In addition, by controlling the vehicle’s turning angle, it can be found that changes in the turning angle also have a significant impact on the head injury criterion (HIC) values of the pedestrian due to the different relative collision positions between the pedestrian and the vehicle. When the direction of the vehicle’s turning is the same as the position of the pedestrian (i.e., the pedestrian is on the left or right in front of the vehicle and the vehicle is turning left or right), the pedestrian’s lower limbs come in contact with the front bumper of the vehicle. Due to the impact speed, the upper body of the pedestrian rotates around the collision point and collides with the engine hood or windshield. As a result, the pedestrian will remain on top of the vehicle’s front end and move together with the vehicle for a longer time. When the direction of the vehicle’s turning is opposite the position of the pedestrian, the front bumper of the vehicle comes in contact with the lower limbs of the pedestrian. The pedestrian rotates around the collision point, but due to the vehicle turning in the opposite direction, the upper body is offset from the front part of the vehicle and does not produce additional collision contact. The pedestrian lands directly on the ground, resulting in a higher risk of head injury.

**Figure 9:**
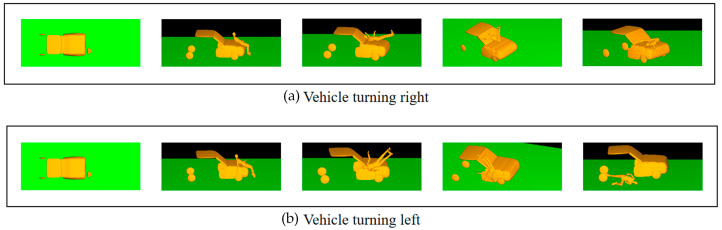
Schematic diagram of collision comparison between vehicles taking different steering measures at the same time: (**a**) Vehicle turning right; (**b**) Vehicle turning left.

This collision process is similar to the collision stage described by Murano [41]. As shown in Figure 9, the collision simulation process caused by the vehicle turning left or right when the pedestrian is located on the right front of the vehicle is illustrated. The collision speed is 40 km/h, and the vehicle’s turning angle is the same in magnitude but opposite in direction. The figure shows a schematic diagram of the collision motion process captured at the same time.

The existence of the collision-turning angle causes the pedestrian to fall on top of the engine hood (windshield) when in contact with the vehicle, and the vehicle produces lateral collision force on the pedestrian, causing the pedestrian to undergo a turning motion and change the landing posture. To address this phenomenon, the pedestrian landing status can be effectively changed by a reasonable adjustment of the turning angle, thereby reducing the risk of pedestrian landing injury.

### 3.3. Impact of Collision Area on Injury

**Figure 10:**
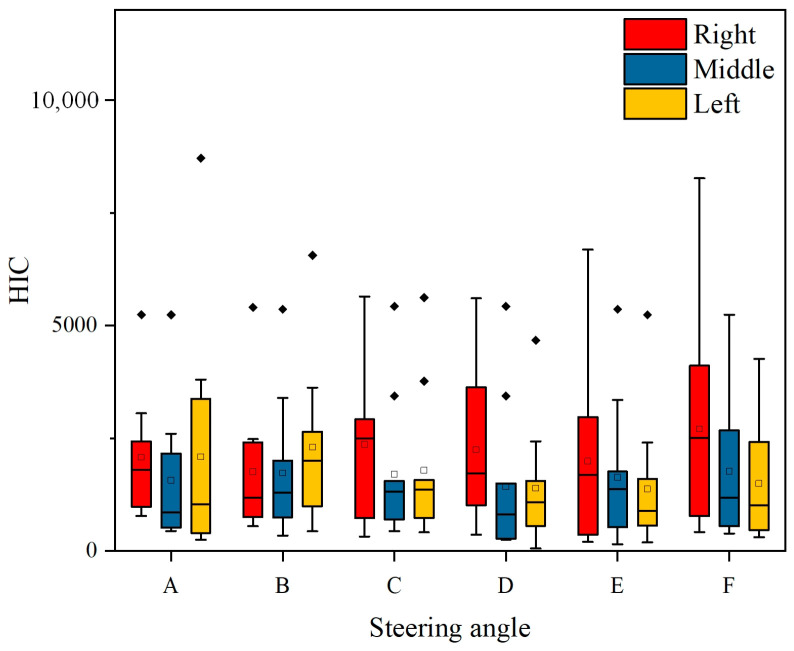
The effect of steering angle on head injury in three collision positions between sedan and pedestrian.

Figure 10 shows the influence of different turning measures by a sedan on the HIC value of head injury when a pedestrian is located at different positions in front of the vehicle. In this figure, A, B, and C represent the vehicle turning right, with turning angles A > B > C; D, E, and F represent the vehicle turning left, with turning angles D < E < F. The three situations represent the pedestrian located in different positions in front of the vehicle, namely, on the right front of the vehicle, in the middle of the front of the vehicle, and on the left front of the vehicle.

It can be observed that as the turning angle increases, the HIC value of head injury to the pedestrian shows different changes depending on the pedestrian’s relative position to the vehicle. When the turning direction of the vehicle is the same as the position of the pedestrian relative to the vehicle, the HIC value of head injury is relatively small (as shown in Figure 10, when the pedestrian is located on the right front of the vehicle, the vehicle turns A, B, and C. When the pedestrian is located on the left front of the vehicle, the vehicle turns D, E, and F). When the turning direction of the vehicle is opposite to the position of the pedestrian relative to the vehicle, it will lead to a larger HIC value for head injury (as shown in Figure 10, when the pedestrian is located on the right front of the vehicle, the vehicle turns D, E, and F. When the pedestrian is located on the left front of the vehicle, the vehicle turns A, B, and C), and the HIC value also shows an increasing trend with the increase in turning angle.

**Figure 11:**
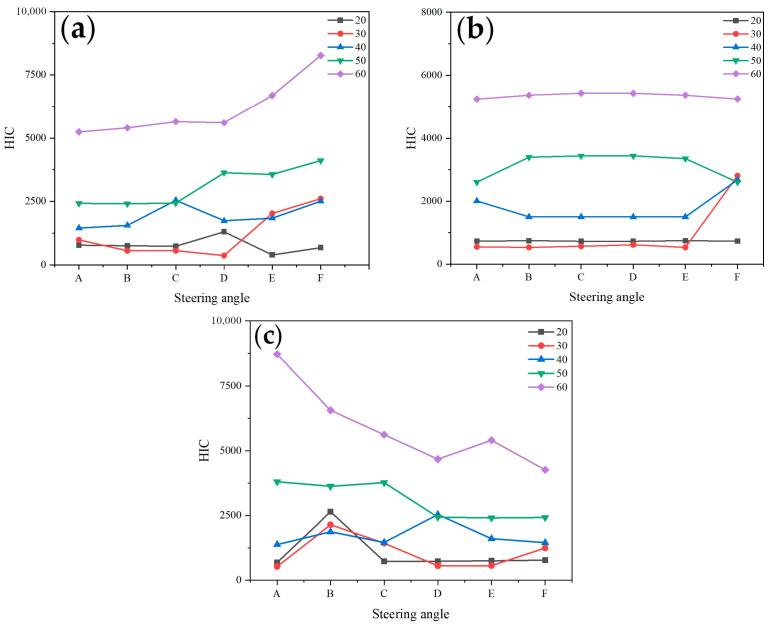
(**a**) The Effect of Steering Angle on HIC when pedestrians on the left side of the vehicle (Sedan); (**b**) The Effect of Steering Angle on HIC when pedestrians on the left side of the vehicle (Sedan); (**c**) The Effect of Steering Angle on HIC when pedestrians are located in the middle region of vehicles (Sedan).

From the simulation results, it can be concluded that for a pedestrian located in the middle area of the front of the vehicle, since the impact of the turning angle on pedestrian injuries is relatively small, no detailed explanation will be given for this type of collision. Figure 11 shows the HIC curve of head injuries caused by vehicles turning for pedestrians at different front positions. By comparing the trend of curves, it can also be determined that due to the different relative positions of the pedestrian and the vehicle, different steering measures of the vehicle will have a significant impact on the head, which is consistent with the above analysis.

### 3.4. The Impact of Vehicle Models on Injury

As shown in Figure 6, it can be seen that the collision damage caused by MPVs is relatively high, while the damage caused by sedans and SUVs is relatively small. The difference in collision damage may be due to the different front-end structures of the vehicles. The bumper height of the MPV is higher than that of the other two models, so when colliding with pedestrians, the collision point between the pedestrian and the vehicle is also higher than other two models. The difference in the contact point between the front end of the vehicle and the pedestrian results in different injury patterns for pedestrians.

In addition, there are significant differences in the inclination angle of the engine hood of the three vehicle models. The MPV engine hood has a larger inclination angle, which results in less time for the pedestrian to stay on the hood after the collision, and the pedestrian is more likely to make direct contact with the ground. The inclination angle of the sedan’s engine hood is smaller, which allows pedestrians to move with the vehicle for a relatively long time, thereby changing their landing posture and causing less damage.

**Figure 12:**
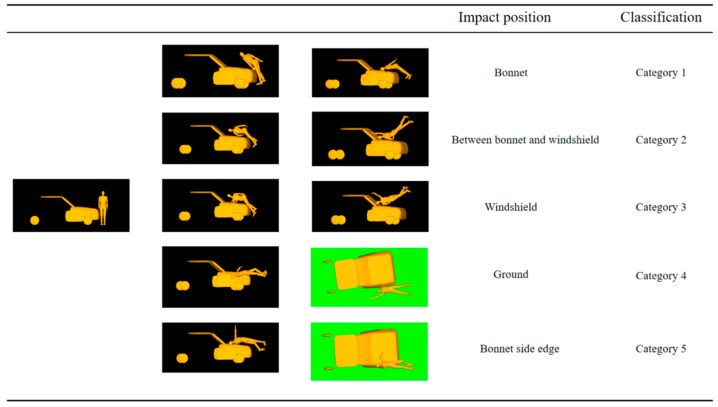
Classification of collision positions.

**Figure 13:**
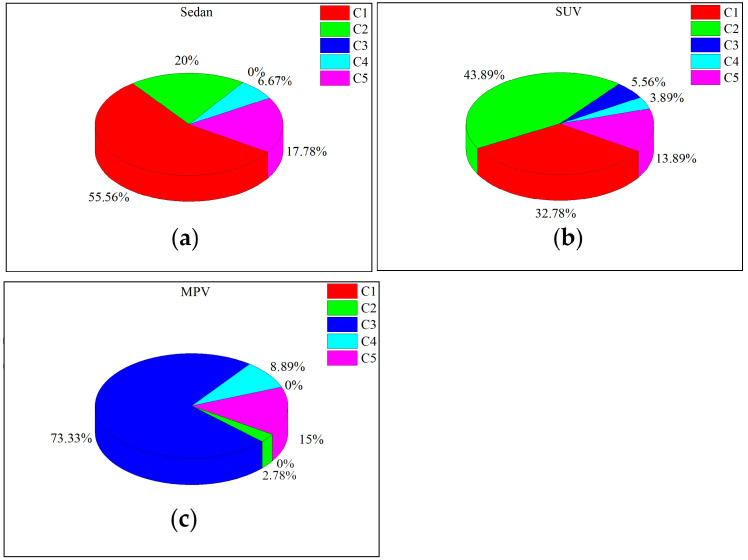
Percentage of collision categories for three vehicle models. (**a**) the proportion of each type of sedan collision; (**b**) the proportion of each type of suv collision; (**c**) the proportion of each type of MPV collision.

This article refers to Crocetta’s analysis method. Figure 12 classifies pedestrian head impact locations. Category 1 is the collision between the head and the engine hood. Category 2 is the collision between the head and the transition area between the hood and windshield. Categories 3, 4, and 5 are collisions between the pedestrian’s head and the windshield, ground, and hood/windshield edges of the vehicle, respectively. The impact location of the head relative to the vehicle is greatly influenced by the shape of the vehicle’s front end. The simulation analysis shows that in the parameter study of three vehicle types and five different speeds, the proportion of each collision category is shown in Figure 13. Most of the collision areas between sedans and SUVs are located in the transition area between the car hood and windshield, which is caused by the longer hood, as shown in the distribution of C1 and C2 in Figure 13. Due to their shorter engine hoods, most of the collision areas are located on the windshield, accounting for 73.33%, as shown in C3 in Figure 13.

### 3.5. Pedestrian Injury

**Figure 14:**
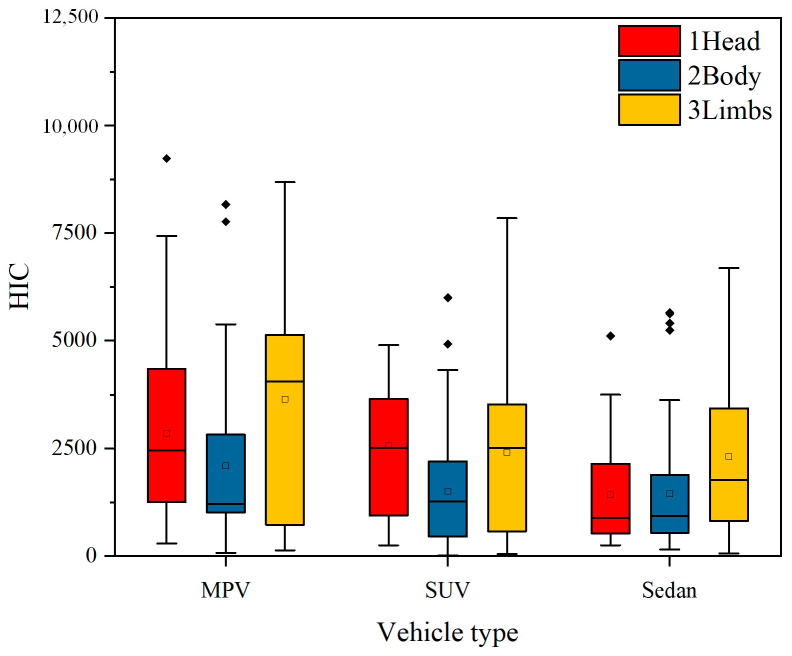
HIC values are caused by three different vehicle models with different landing methods.

**Figure 15:**
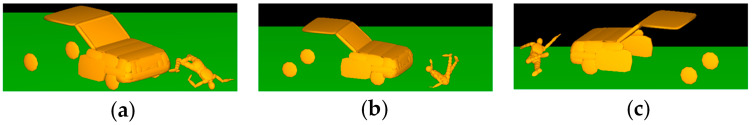
Pedestrian landing method: (**a**) head landing; (**b**) torso landing; (**c**) fall on all fours.

This study explores the impact of vehicle steering on pedestrian injury by comparing various collision parameters. By comparing and analyzing, it can be concluded that collision speed, turning angle, and vehicle type have a significant impact on pedestrian injuries. Pedestrian head injuries, is mainly affected by the pedestrian’s landing posture. When the pedestrian’s head lands first due to a collision, it will generate a large center of gravity acceleration (CG) and HIC value, which will cause significant damage to the head, as shown in Figure 14. When the trunk or limbs land first, it can provide some protection for the pedestrian’s head (Figure 15). Through data analysis, it can be concluded that regardless of the vehicle type, when colliding with pedestrians, if the pedestrian’s head hits the ground first, it will cause a higher HIC value. If a pedestrian lands on the ground with their trunk or limbs first after a collision, it can have a certain buffering effect on the head collision and to some extent alleviate the damage caused to the head. However, due to the soft limbs, the buffering effect on the head collision is not as good as in the case of the trunk landing first.

## 4. Discussion

### 4.1. Sensitivity Analysis

**Figure 16:**
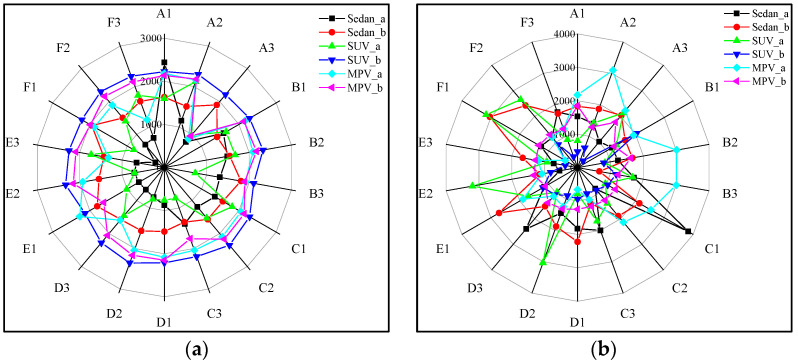
(**a**) radar chart diagram of peak acceleration of pedestrian-vehicle collision; (**b**) radar chart diagram of peak acceleration of human ground collision.

Reducing pedestrian injuries in pedestrian-vehicle collision accidents using feasible control methods is a significant challenge. Through our research, we have found that pedestrian injuries are influenced by vehicle steering, particularly in terms of head injuries. Figure 16 is a radar chart showing the peak acceleration generated by pedestrian collisions. Figure A represents the radar chart of peak acceleration generated by vehicle-to-pedestrian head collisions under different steering measures. Its peak acceleration range is 0–3000. Figure B represents the radar chart of peak acceleration generated by pedestrian head-to-ground collisions. Its peak acceleration range is 0–4000. A-F represents the six steering angles, 1–3 represent the relative positions of the human vehicle, while a and b represent the relative orientations of the human vehicle. By observing the different fluctuations in the data curves in the graph, we can conclude that for a primary collision (vehicle-to-pedestrian collision), the acceleration generated is less affected by the steering angle, with a relatively small range of peak acceleration variation. However, for a secondary collision (pedestrian-to-ground collision), the peak acceleration generated is more influenced by the steering, indicating that the steering measures have a greater impact on reducing the peak acceleration in pedestrian-to-ground collisions compared to pedestrian-to-vehicle collisions.

This phenomenon occurs because, during the initial collision between the vehicle and the pedestrian, there is contact between the pedestrian’s lower limbs and the front bumper of the vehicle. This contact causes the pedestrian to rotate around the point of impact, resulting in the pedestrian landing on the vehicle’s hood. As a result, there is contact between the pedestrian’s head and the engine hood or windshield.

In this study, although vehicle steering may cause differences in the initial collision position between the pedestrian’s head and the vehicle, overall, the head still collides with the front windshield/hood of the vehicle. Therefore, the impact of the steering angle on the collision is relatively small. After the initial contact between the person and the vehicle, the vehicle is still in the deceleration phase. The pedestrian moves along with the vehicle. When the vehicle stops moving, the pedestrian lands due to the inertia generated by the collision. Due to the differences in the vehicle’s steering angle and the pedestrian’s position, there will be variations in the landing time and posture of the pedestrian, as shown in Figure 8. This leads to significant differences in the extent of injuries.

### 4.2. Correlation between Collision Parameters and Damage

This study classified and organized the collision data obtained from simulations. When the pedestrian is in the middle area of the vehicle’s front end, the steering angle has a relatively small impact on head injuries. However, when the pedestrian is on the left or right side of the vehicle’s front end, the vehicle’s steering actions can have a significant impact on head injuries. Therefore, we focused on analyzing the correlation in the cases where the pedestrian is on the sides. We evaluated the correlation between the Head Injury Criterion (HIC) values and collision speed, pedestrian orientation, steering angle, and vehicle shape to guide the assessment of head injury severity based on HIC values.

The Spearman method in SPSS was employed, see Table 4 and Table 5. Table 4 and Table 5, respectively, present the correlation analysis of various parameters when the pedestrian is located in the right front and left front of the vehicle. Based on the analysis in Table 4, it is evident that there is a significant correlation between the Head Injury Criterion (HIC) value of the pedestrian’s head injury and the vehicle’s steering angle, collision speed, and the contact area of the pedestrian-vehicle collision. Among them, the steering angle is negatively correlated with the HIC value, meaning that as the steering angle of the vehicle increases, the HIC value of the pedestrian’s head injury will relatively decrease. On the other hand, the collision speed and the frontal area of the vehicle are positively correlated with the HIC value. Different collision speeds and collision areas will result in variations in the HIC value.

By comparing Table 4 and Table 5, it can be observed that in Table 4, there is a negative correlation between the steering angle and the HIC value. However, in Table 5, the steering angle is positively correlated with the HIC value. This situation is caused by the different relative positions of pedestrian-vehicle collisions. When the pedestrian is located in the right front of the vehicle, the vehicle takes steering actions. For the convenience of statistical analysis, the positive direction is defined as steering angles A, B, and C (right turn, where angle A > B > C); while the negative direction is defined as steering angles D, E, and F (left turn, where angle D < E < F). Based on the data analysis, it can be concluded that as the vehicle’s steering angle changes from A to F, there is a decreasing trend in the numerical values of the steering angle. Through correlation analysis, it is found that the Head Injury Criterion (HIC) value of the pedestrian’s head injury will increase. This conclusion is consistent with the conclusion in Section 3.3.

### 4.3. Regression Analysis and Comparative Validation

Based on the previous analysis, it is evident that the head injuries of pedestrians are influenced by the steering angle of the vehicle and the relative position difference between the pedestrian and the vehicle. Therefore, the simulated accident cases can be classified and discussed based on the different steering directions and relative positions between the pedestrian and the vehicle.

**Figure 17:**
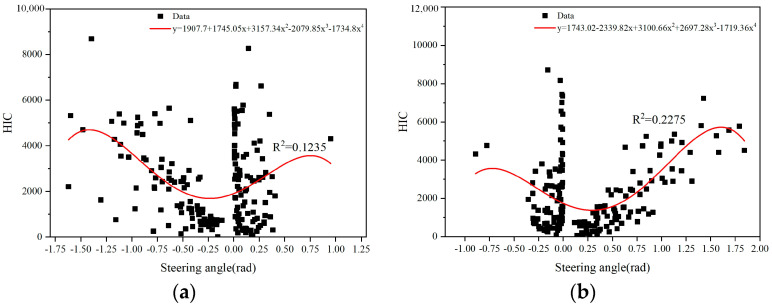
(**a**) Steering angle and HIC fitting curve (The pedestrian is located in the right front of the vehicle); (**b**) Steering angle and HIC fitting curve (The pedestrian is located in the left front of the vehicle).

However, due to the varying collision speeds, we applied the same braking measure during the case simulation. As a result, there may be differences in the vehicle’s steering angle due to the braking action. Regarding this, we conducted a nonlinear fitting analysis between the simulated final steering results and the HIC values of head injury for pedestrians. The results are presented in Figure 17.

Figure 17a shows the fitting relationship between the vehicle’s steering angle and the HIC value of head injury when the pedestrian is located in the right front of the vehicle. By observing the curve, it can be seen that as the degree of right turn of the vehicle (rad < 0) decreases, the HIC value of head injury also decreases. On the other hand, when the vehicle takes a left turn (rad > 0), the HIC value of pedestrian head injury tends to increase with the increasing degree of steering. Figure 17b shows the fitting relationship between the vehicle’s steering angle and the HIC value of head injury when the pedestrian is located in the left front of the vehicle. In this case, the HIC value of head injury decreases as the degree of right turn of the vehicle (rad < 0) decreases, and it increases as the degree of left turn of the vehicle (rad > 0) increases.

The results here further validate the conclusion in Section 3.3. Through the analysis of two fitting curves, it can be observed that when the vehicle’s steering degree is around 0.28 rad, the resulting head injury to the pedestrian is relatively low. (This holds when the pedestrian is located in the right front of the vehicle and a right turn is taken, as well as when the pedestrian is located in the left front of the vehicle and a left turn is taken).

**Figure 18:**
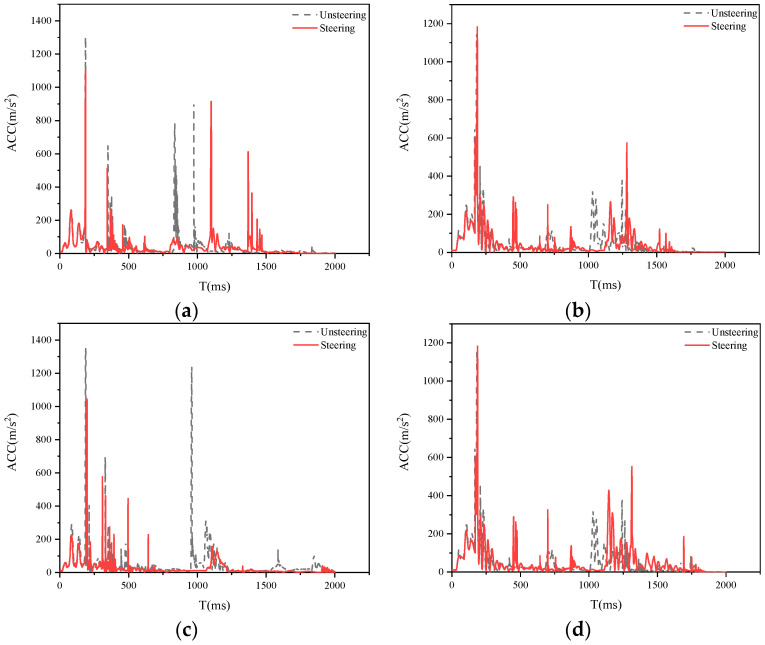
Collision acceleration curve generated by Sedan turning at 0.28 rad. (**a**) Right-a; (**b**) Right-b; (**c**) Left-a; (**d**) Left-b.

**Figure 19:**
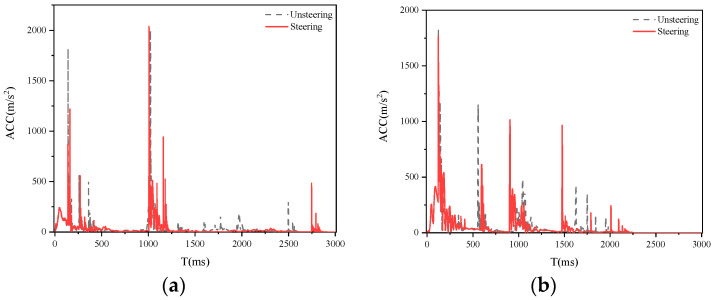

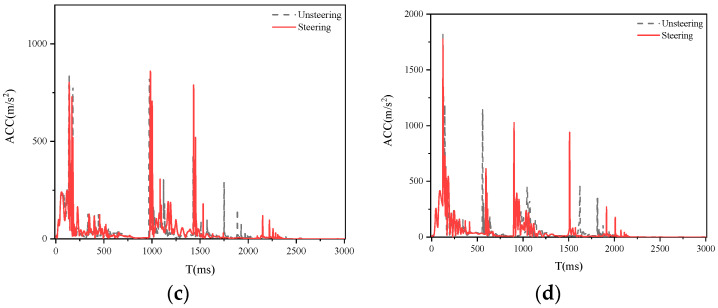
Collision acceleration curve generated by SUV turning at 0.28 rad. (**a**) Right-a; (**b**) Right-b; (**c**) Left-a; (**d**) Left-b.

**Figure 20:**
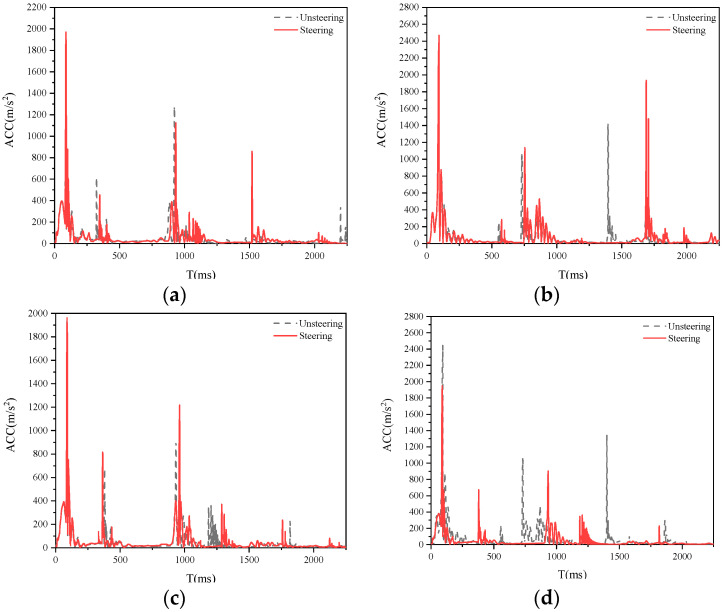
Collision acceleration curve generated by MPV turning at 0.28 rad. (**a**) Right-a; (**b**) Right-b; (**c**) Left-a; (**d**) Left-b.

Figure 18, Figure 19 and Figure 20 show a comparison of the damage caused by a collision between a sedan, SUV, and MPV with a pedestrian at speeds of 20, 30, and 40 km/h, with and without 0.28 rad steering measures. From the graph, it can be observed that the steering measures taken by SUV models have the most significant effect on reducing collision acceleration, followed by sedans and MPVs, which may be due to the following reasons. The previous nonlinear fitting analysis conducted a unified analysis of all the analysis results of this study and did not classify and fit curves according to different vehicle models, resulting in insufficient compatibility between the fitted curves and MPV models. Subsequent research will classify and fit different vehicle models to further explore the optimal steering angle for each type of vehicle.

The SUV model has good adaptability, further verifying the previous simulation results. Reasonable turning measures based on the different motion states of vehicles can effectively change the landing posture of pedestrians, thereby reducing pedestrian head injuries. When a vehicle collides with a pedestrian, adopting the same turning direction as the pedestrian’s position can allow the pedestrian to stay above the vehicle’s front for a long time and move together with the vehicle, effectively alleviating head injuries. When the vehicle turns in the opposite direction from the pedestrian’s position, the pedestrian’s upper body is staggered from the front of the vehicle after the collision without generating additional collision contact. The pedestrian directly collides with the ground, resulting in higher head injuries.

### 4.4. Formatting of Mathematical Components

This study aims to analyze the impact of vehicle steering measures on the severity of pedestrian injury in pedestrian-vehicle collision accidents. This study has certain limitations. The first limitation is the lack of parameters for actual collisions between turning vehicles and pedestrians, and the absence of a standardized quantification for vehicle steering. Additionally, suitable collision data for this specific scenario were not found in published literature. Therefore, in this study, the researchers reconstructed PC accidents using existing real-world traffic accident cases in the laboratory. They obtained the approximate motion process of the turning vehicle colliding with the pedestrian during the accident. Then, the researchers used MADYMO software to reconstruct a multi-body model and conduct simulation calculations to analyze pedestrian injuries.

The second limitation of this study is that it did not consider the biomechanical characteristics of the human body, such as the properties of skin and tissue. This study focused on the impact of vehicle collision parameters (vehicle steering) on pedestrian injuries. Therefore, the analysis of collision injuries was conducted using a single pedestrian model. The emphasis of the study was not on the variations in pedestrian body types, which is why the discussion on the differences in pedestrian body types was not included.

## 5. Conclusions

This study utilized MADYMO simulation experiments to investigate the impact of vehicle steering evasive maneuvers on head injuries in pedestrians during sudden emergencies. Through analysis of six specific steering angles, five collision speeds, three vehicle types, three collision positions, and two pedestrian orientations, the following conclusions can be drawn within the scope of the study:

1. This study used PC-Crash to reconstruct collision accidents and conducted collision simulations using MADYMO. By defining the initial velocity of the vehicle, the vehicle’s steering process was achieved by assigning lateral velocity and rotational angular velocity. Different collision parameters were set to evaluate the risk of head injury to pedestrians under different vehicle steering conditions.

2. In the face of sudden pedestrian crossing emergencies, the implementation of steering evasive maneuvers by vehicles can effectively reduce the risk of head injuries to pedestrians. Specifically, when the vehicle adopts a steering angle of approximately 0.28 rad, the resulting head injury to pedestrians is generally low. Furthermore, as the collision speed of the vehicle increases, the head injury criterion (HIC) value for head injury to pedestrians also increases. Among the different vehicle types considered, the MPV has the greatest impact on head injuries.

3. Due to different relative positions between pedestrians and vehicles, different steering maneuvers by vehicles can lead to varying impact outcomes. When the direction of the vehicle turning is the same as the position of the pedestrian, it can result in the pedestrian being trapped above the vehicle’s front and moving together with the vehicle for a longer time after the collision. This can effectively reduce the risk of head injuries to the pedestrian. However, when the direction of vehicle steering is opposite to the position of the pedestrian, the pedestrian directly collides with the ground, leading to higher levels of head injury.

4. By comparing the differences among the three collision vehicle types, it can be observed that due to the different front-end structures, the MPV has the highest inclination angle of the engine hood, resulting in a higher head collision position compared to other two vehicle types. As a result, the MPV causes relatively higher collision injury values at any steering angle, while the sedan and SUV cause relatively smaller injury values.

In the future, the findings of this study can be applied to the design of practical vehicle safety systems. Specifically, this study determined the optimal steering angle (approximately 0.28 rad) in an emergency collision avoidance scenario. This finding can be used as a reference for automobile manufacturers when designing intelligent driver assistance systems, especially when developing emergency collision avoidance algorithms. By integrating this steering strategy into an autonomous driving system to automatically adjust the steering angle, pedestrian injuries can be minimized in unavoidable collision events. It can also provide relevant data to support further refinement of emergency collision avoidance systems and may lead to the development of new design standards. In addition, the simulation and optimization methods established in this study can be applied to other driving scenarios to enhance pedestrian safety. In addition to specific steering evasive maneuvers, it is also possible to consider risk avoidance strategies that combine with braking measures and consider different steering control strategies for different scenarios.

## Figures and Tables

**Table 1:** Front structure parameters and pedestrian physical parameters.

	Vehicle			Pedestrian	
Parameters	Sedan	SUV	MPV	50th percentile adult male	
H (m)	0.824	0.994	0.827	Height (m)	1.74
L (m)	1.13	0.979	0.573	Weight (kg)	75.7
θ (°)	30	34	46	Height of C.G (m)	0.958
α (°)	11	8	20		

**Table 2:** Steering angle setting parameter.

Steering Angle	Transverse Velocity (Vy)	Palstance (w)
A	−1.8 m/s	−0.9 rad/s
B	−1.2 m/s	−0.65 rad/s
C	−0.8 m/s	−0.4 rad/s
D	0.8 m/s	0.4 rad/s
E	1.2 m/s	0.65 rad/s
F	1.8 m/s	0.9 rad/s

**Table 3:** Pedestrian-vehicle crash parameters.

Pedestrian	Orientation	Vehicle Type	Collision Area	Collision Speed	Steering Angle
50th percentile adult male	Back	Sedan	Right	20 km/h	A
	Side	SUV	Middle	30 km/h	B
		MPV	Left	40 km/h	C
				50 km/h	D
				60 km/h	E
					F

**Table 4:** The correlation coefficient between HIC and collision variables (Right).

	Steering Angle	Orientation	Collision Speed	HIC	Collision Area	Vehicle Type
Steering angle	1					
Orientation	0.0037	1				
Collision speed	−0.312	0.000	1			
HIC	−0.192 **	−0.263 **	0.802 **	1		
Collision area	0.508 **	0.184 *	−0.023	−0.027	1	
Vehicle type	−0.042	0.000	0.000	0.157 *	0.223 **	1

** Correlation is significant at the 0.01 level (2-tailed). * Correlation is significant at the 0.05 level (2-tailed).

**Table 5:** The correlation coefficient between HIC and collision variables (Left).

	Steering Angle	Orientation	Collision Speed	HIC	Collision Area	Vehicle Type
Steering angle	1					
Orientation	0.004	1				
Collision speed	0.328	0.000	1			
HIC	0.256 **	−0.318 **	0.812 **	1		
Collision area	−0.448 **	−0.098	0.025	0.208 **	1	
Vehicle type	0.077	0.000	0.000	0.199 **	0.400 **	1

** Correlation is significant at the 0.01 level (2-tailed).

## Data Availability

The study did not report any data.
